# Exploring the immunomodulatory effects and mechanisms of Xinjiang fermented camel milk-derived bioactive peptides based on network pharmacology and molecular docking

**DOI:** 10.3389/fphar.2022.1038812

**Published:** 2023-01-04

**Authors:** Yuxing Wang, Zhuangzhuang Liang, Fang Shen, Wenting Zhou, Tabusi Manaer, Didaier Jiaerken, Xinhua Nabi

**Affiliations:** ^1^ Department of Pharmacology, Xinjiang Medical University, Urumqi, China; ^2^ State Key Laboratory of Natural Medicines, Jiangsu Key Laboratory of Drug Discovery for Metabolic Diseases, China Pharmaceutical University, Nanjing, China; ^3^ Xinjiang Uygur Autonomous Region Institute for Drug Control, Urumqi, China

**Keywords:** fermented camel milk from Xinjiang, bioactive peptides, immune regulation, network pharmacology, molecular docking

## Abstract

**Purpose:** Fermented camel milk from Xinjiang is rich in probiotics and has immunomodulatory effects as an important source of bioactive peptides. However, it is not clear whether it is the probiotic or the bioactive peptide that acts. The present study aimed to extract and identify bioactive peptides from fermented camel milk in Xinjiang and investigate their immunomodulatory effects and mechanisms based on network pharmacology and molecular docking.

**Methods:** Four probiotic bacteria were used to ferment the fresh camel milk and the bioactive peptides were extracted and isolated by ultrafiltration and column chromatography. Network pharmacology predicts targets and pathways of action. GeneCards and OMIM-GENE-MAP database were used in order to search disease target genes and screen common target genes. Then we used STRING web to construct a protein-protein interaction (PPI) interaction network of the common target protein. The key targets were analyzed by GO (Gene Ontology) and KEGG (Kyoto Encyclopedia of Genes and Genomes) analysis through the David database. The "drug (bioactive peptide)-disease-targets-pathway" network was established and molecular docking was used for prediction.

**Results:** Two fractions were obtained by UV spectrophotometer; whey acidic protein, α-lactalbumin, and peptidoglycan recognition protein 1 were the main protein-like components of Xinjiang fermented camel milk-derived bioactive peptides. The repeat sequence of peptidoglycan recognition protein 1 was selected and then seven bioactive peptides were obtained. Bioactive peptides had 222 gene targets, anti-inflammatory diseases had 2598 gene targets, and immune regulation had 866 gene targets, the intersection of which was 66 in common gene targets. Gene ontology and KEGG analysis reveals that bioactive peptides mainly play a vital role in the signaling pathways of lipid and atherosclerosis, pathways in cancer. The molecular docking results showed that the seven bioactive peptides bound well to the top four scoring proteins.

**Conclusion:** The immunomodulatory and anti-inflammatory effects and mechanisms of Xinjiang fermented camel milk-derived bioactive peptides were initially investigated by network pharmacology and molecular docking, providing a scientific basis for future studies.

## 1 Introduction

The immune system is a critical defense system for the organism (Poles et al., 2021). Immunomodulatory abnormalities often lead to autoimmune tolerance disorders and autoimmune diseases, and understanding their underlying mechanisms could provide potential new targets for more effective therapies (Giat et al., 2017). It is estimated that 7.6%–9.4% of the world population has various types of autoimmune diseases (Cooper et al., 2009), which makes it the third most common chronic disease after cardiovascular diseases and cancer (Zhai and Yang, 2022). In recent years, the incidence of immunological diseases has been on the rise (Aaron et al., 2015), seriously affecting the quality of life of the workforce and patients, even threatening their lives.

Small molecule drugs and targeted biologic are the mainstays of treatment (Cheng and Li, 2021). The increasing number of patients with autoimmune diseases calls for new therapeutic approaches. These immune checkpoint molecules, such as CTLA-4 and PD-1, have been shown to be involved in the development of autoimmune diseases and could be targeted for treatment (Huang et al., 2019). Changes in immune function are also associated with intestinal flora, and in particular, intestinal immunity is linked to the development of many chronic inflammatory diseases, such as diabetes (Scheithauer et al., 2020), cardiovascular disease (Zhou et al., 2020) and tumors (Qiu et al., 2021). Probiotics and bioactive peptides also play an adjuvant therapeutic role in their treatment process (Wong et al., 2022; Wang et al., 2020a; Wang et al., 2020a). Probiotics have also been reported as an effective preventive and therapeutic strategy for the treatment of allergic/autoimmune diseases (Isolauri et al., 2012; Liu et al., 2018). At the same time, bioactive peptides are receiving increasing attention due to their involvement in various biological processes (Chakrabarti et al., 2018). Antimicrobial peptides, such as Cathelicidins, not only modulate inflammatory responses and B cell function but also attenuate autoimmune diseases (Sun et al., 2016; Sun et al., 2015). The production of bioactive peptides is caused by protein degradation, their lower molecular weight, easier absorption by the body and various biological properties such as hypotensive, antioxidant, antibacterial, anti-inflammatory, chelating, and other effects (Jakubczyk et al., 2020). Milk protein is considered an important source of bioactive natural peptides (Vargas-Bello-Pérez et al., 2019). Conventional fermented foods containing lactic acid bacteria have been shown to have beneficial effects on human health, some of which are associated with protein-derived products including bacteriocins and bioactive peptides (Venegas-Ortega et al., 2019).

Scholars have found that the prevalence of metabolic syndrome in the Kazakh population is lower than that of other ethnic groups in Xinjiang, China, which is closely related to their long-term daily habit of using contain bioactive peptides products such as traditional fermented cheese whey and fermented camel milk (Wang et al., 2010; Li et al., 2012; Yan et al., 2015). In our previous work, we found that traditional fermented cheese whey has anti-atherosclerotic and anti-inflammatory roles in rats (Nabi et al., 2007) and anti-atherosclerotic effects in rabbits (Nabi et al., 2016), and found that probiotic fermented camel milk has significant hypoglycemic potential in rats models of type 2 diabetes mellitus (Manaer et al., 2015). Also we found that the composite probiotics from fermented camel milk has anti-diabetic effect in *db/db* mice (Wang et al., 2020b). Fourteen probiotics were isolated from fermented camel milk in Xinjiang (Ma et al., 2010; Liu et al., 2017), and four of them were found to alleviate the immune deficiency in mice caused by cyclophosphamide (Yusufu et al., 2022) and to exert anti-tumor effects by adjusting the CD^4+^/CD^8+^ cell ratio and increasing the secretion of Th1-type cytokines (Xiao et al., 2017). We also found that bioactive peptides extracted from fermented camel milk from Xinjiang had anti-inflammatory and immunomodulatory functions (Zhang et al., 2015) and promoted the expression of IFN-γ (Wulazibieke et al., 2014).

**FIGURE 1:**
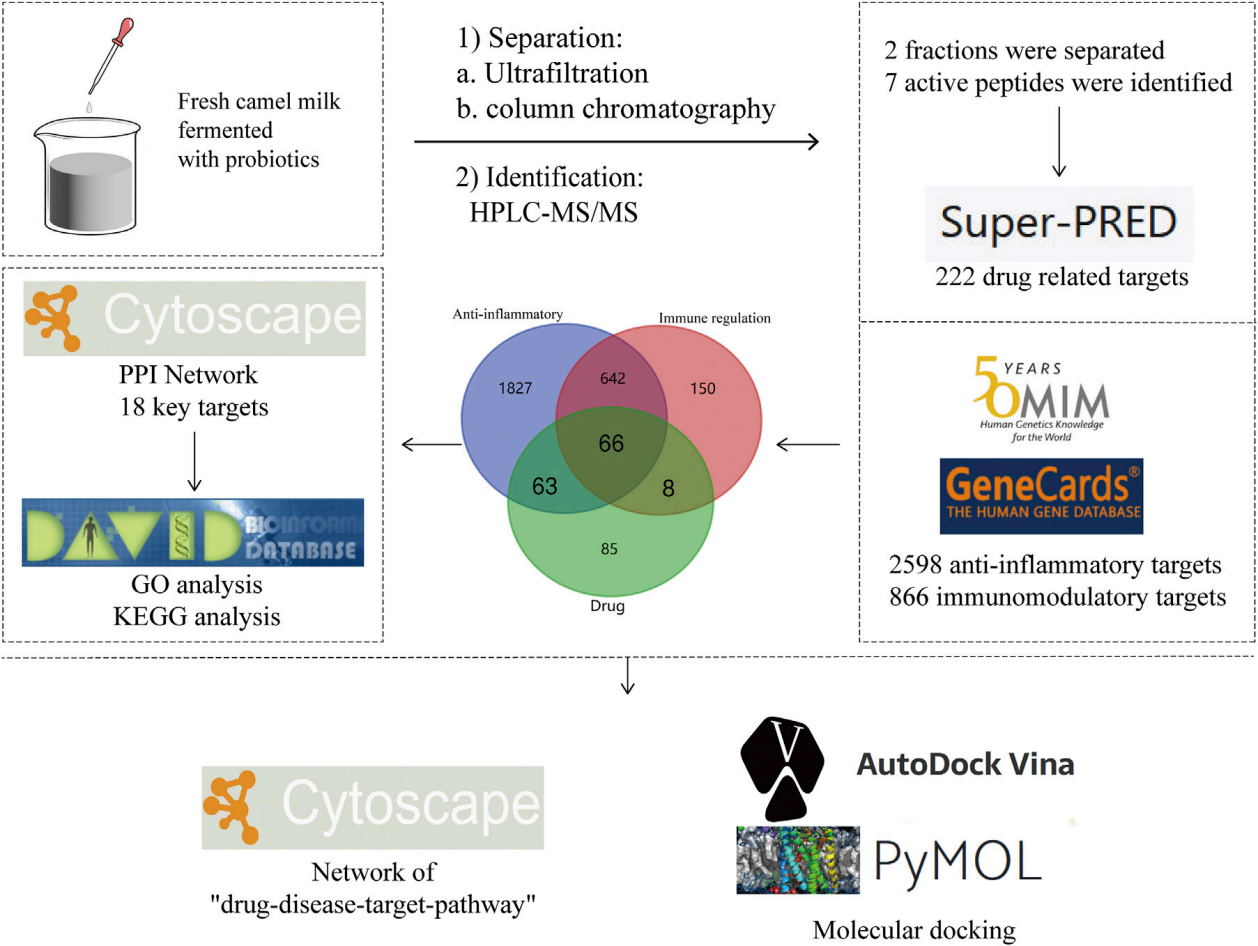
The procedure for this study.

In this study, we fermented fresh camel milk with four probiotic bacteria (*Issatchenkia orientali*, et al.) (Yusufu et al., 2022; Xiao et al., 2017). Bioactive peptides were extracted and isolated from the fermented camel milk and identified by HPLC-MS/MS. Through preliminary studies, it was shown that this bioactive peptide has immunomodulatory and anti-inflammatory effects, but the targets and pathways of action are not clear. The potential targets and signaling pathways of the bioactive peptide with immunomodulatory and anti-inflammatory effects were predicted by using the network pharmacology method combined with molecular docking, which provided a theoretical basis for further large data integration experiments. The procedure for this study is shown in Figure 1.

## 2 Materials and methods

### 2.1 Extraction and identification of fermented camel milk-derived bioactive peptides

The fresh camel milk was centrifuged at 3,000 r/min for 30 min and repeated three times, the upper-fat layer was discarded and pasteurized. The fermented camel milk was centrifuged at 3,000 r/min for 15 min repeated three times, and collected whey. The whey was ultrafiltered using a nanofiltration membrane with a molecular retention value of 30 ku, and the filtrate was concentrated and freeze-dried for 24 h to obtain the crude lyophilized powder. The crude lyophilized powder was separated by Sephadex-50 gel column chromatography, and one tube was collected every 3 ml. The absorption value was detected at 214 nm (Baheti et al., 2014) using a UV spectrophotometer, and the eluates with the same peak time were combined and freeze-dried for 24 h. The bioactive peptides obtained were identified by the HPLC-MS\MS method, which was performed by Beijing Protein Innovation Co., Ltd.

### 2.2 Target screening for bioactive peptides and diseases

The 2D structures of the identified bioactive peptides were drawn using MarvinSketch 22.11 software (Li et al., 2022) and the obtained bioactive peptides were subjected to target prediction through the Super PRED (https://prediction.charite.de/) database. Then the GeneCards (https://www.genecards.org/) and OMIM-GENE-MAP (https://omim.org/search/advanced/geneMap) databases were then screened using “immunomodulatory”" and “anti-inflammatory” as keywords to obtain disease-related targets (set Score > 10 in GeneCards for both keywords). Gene targets at the intersection of bioactive peptide immunomodulation and anti-inflammatory diseases were obtained through the online platform of Venn diagram mapping (http://bioinformatics.psb.ugent.be/webtools/Venn/).

### 2.3 Network constructions for PPI

The intersecting gene targets were entered into the String web (https://www.string-db.org), set to “"homo sapiens” species, and the network relationship data of the target protein interactions were obtained, and the PPI was mapped using Cytoscape 3.9.0 software. Network diagrams were created using Cytoscape 3.9.0 and key targets were screened using the Network Analyzer tool (Chen et al., 2022).

### 2.4 Gene function and pathway enrichment

GO and KEGG enrichment analyses of key targets were performed through the David database (https://david.ncifcrf.gov/), set at *p* < .05, to obtain the functions and pathways of key targets.

### 2.5 Network construction

The results of the intersection of bioactive peptide and disease key targets and KEGG pathway enrichment analysis were collected and imported into Cytoscape 3.9.0 software to create a “drug(bioactive peptide)-disease-target-pathway” network.

### 2.6 Molecular docking

The experiments used molecular docking to validate interactions between bioactive peptides and predicted targets. The 2D structures of the bioactive peptides were converted to 3D structures using MarvinSketch 22.11. The 3D structures of key target proteins were obtained using the PDB (protein data bank) database (http:/www.rcsb.org/pdb). Then the operations such as removal of water molecules, hydrogen addition, and setting the small molecule ligand flexible bonds to rotatable were performed by AutoDockTool 1.5.7 (Zhu et al., 2021). Finally, the docking process was performed using AutoDock Vina 1.1.2 and molecular binding energies were calculated.

### 2.7 Molecule dynamics

Based on the experimental results, the molecular dynamics simulation of bioactive peptide 2/STAT3 and bioactive peptide 5/CASP3 complex was performed using Desmond software (Liu et al., 2021). A reasonable number of Na^+^ and Cl^−^ ions were added to keep the system neutral, and the final generation phase of 100 ns was achieved (Chen et al., 2020).

## 3 Results

### 3.1 Results of extraction and isolation of bioactive peptides

**FIGURE 2:**
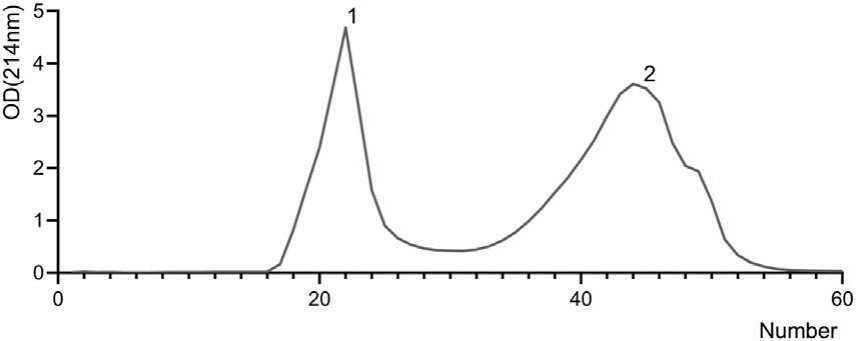
The fractions obtained by gel chromatography. Column chromatography: one tube per 3 ml was collected, 60 tubes in total.

Fresh camel milk was fermented with a mixture of four probiotics to separate the whey, and the crude product with a molecular weight of less than 30 ku was obtained by ultrafiltration. The crude product was then separated on a Sephadex G-50 gel column and detected at 214 nm with a UV spectrophotometer to obtain two fractions (Figure 2).

### 3.2 Identification of bioactive peptides and duplicate peptides

The two bioactive peptides were identified by HPLC-MS/MS and 30 proteins were identified, of which whey acidic protein, α-lactalbumin and peptidoglycan recognition protein 1 were the main protein-like components. It was shown that peptidoglycan recognition protein 1 has various biological activities such as bactericidal, antitumor, and immunomodulatory activities (Nylund et al., 2018). The sequences duplicated with peptidoglycan recognition protein 1 were selected, and a total of seven bioactive peptides were obtained after de-duplication (Table 1).

**TABLE 1:** Seven bioactive peptides.

Num	Peptide sequence	Mr
1	DVQPTLSPGDR	1183.6
2	AAQNLLACGVALGALR	1596.9
3	LYEIIQTWSHYR	1607.8
4	YVVVSHTAGSHCDTPASCAQQAQNVQSYHVR	3456.6
5	EDPPACGSIVPR	1296.6
6	GHRDVQPTLSPGDR	1533.8
7	GAHAGPTWNPISIGISFMGNYMNR	2606.2

### 3.3 Targets related to the immunomodulatory effects of bioactive peptides

**FIGURE 3:**
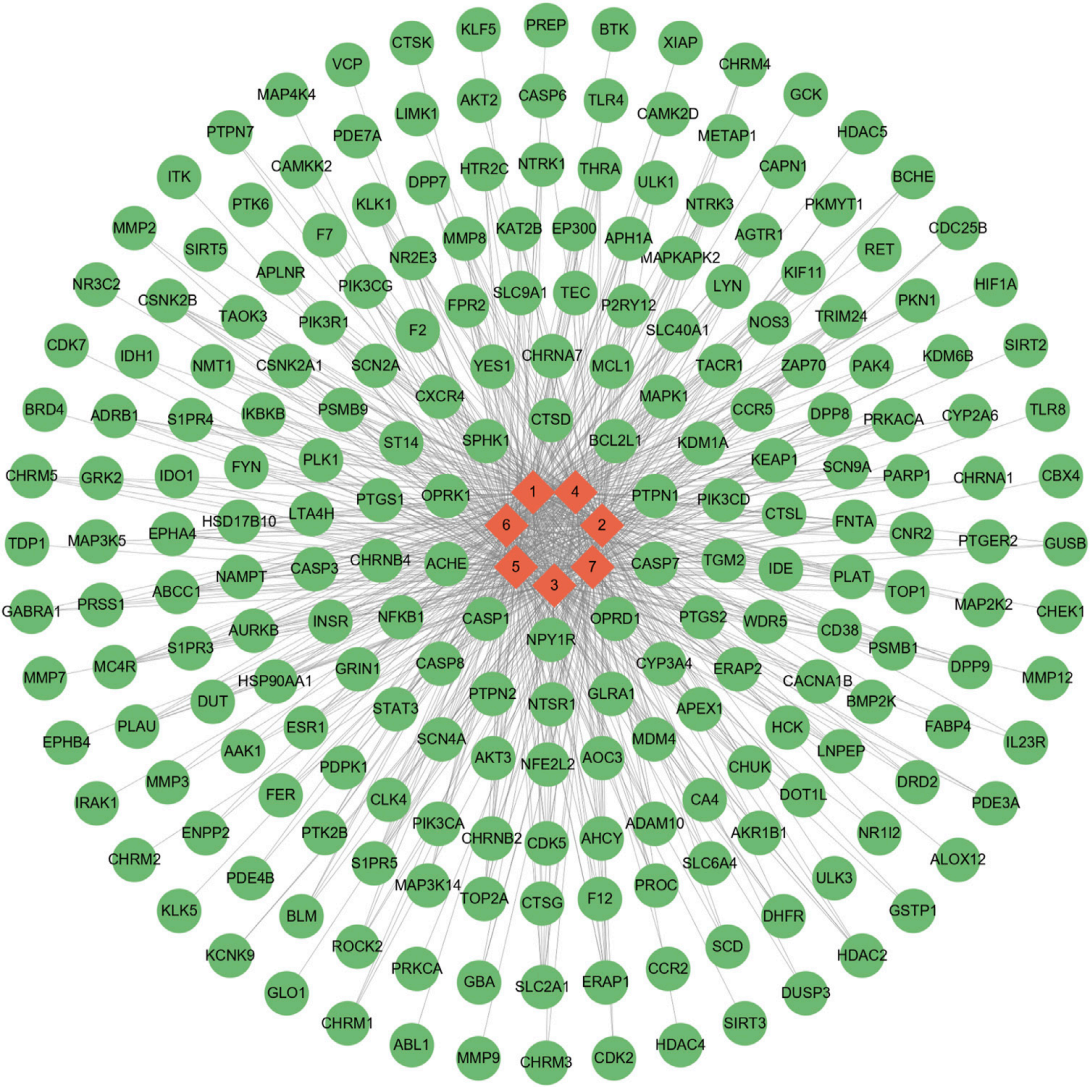
network of “drug (bioactive peptide)-target.” Red nodes drug; green nodes represent target and the line segments represent the relationship between the two.

**FIGURE 4:**
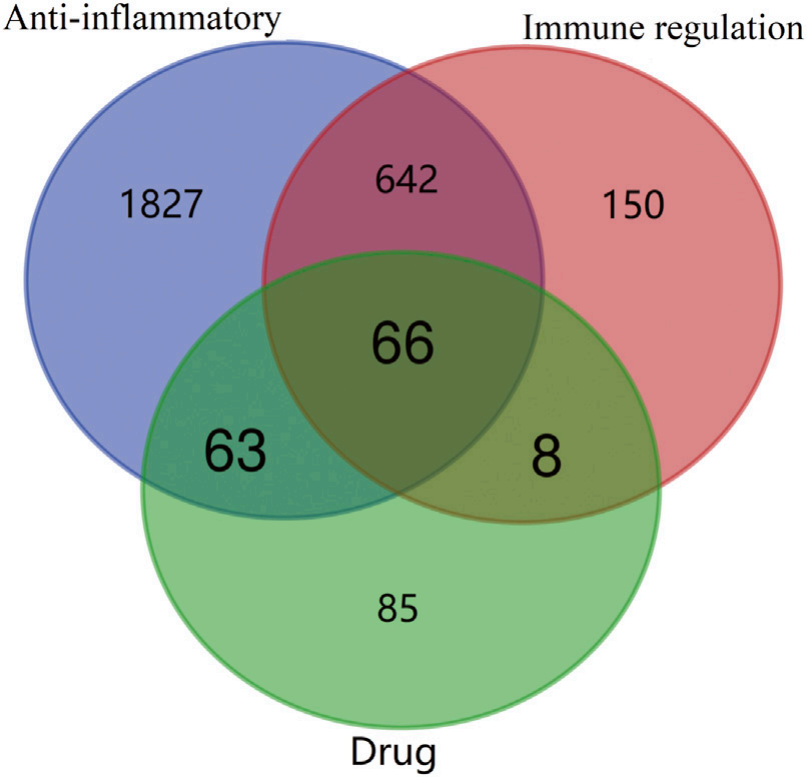
Venn diagram of drug (bioactive peptide) and disease intersection targets.

The seven bioactive peptides were retrieved from the Super PRED database and 222 potential targets were identified. The bioactive peptides and potential targets were imported into Cytoscape 3.9.0 to construct the “bioactive peptide-target” network (Figure 3). The GeneCards database and OMIM-GENE-MAP database were used to screen potential disease targets, and duplicate values were combined and removed, resulting in a total of 2,598 anti-inflammatory gene targets and 866 Immune regulation gene targets. The crossover of drug targets and disease targets was performed using the online platform of Venn diagram mapping, and a total of 66 crossover targets were obtained (Figure 4).

### 3.4 PPI network construction and key target screening

**FIGURE 5:**
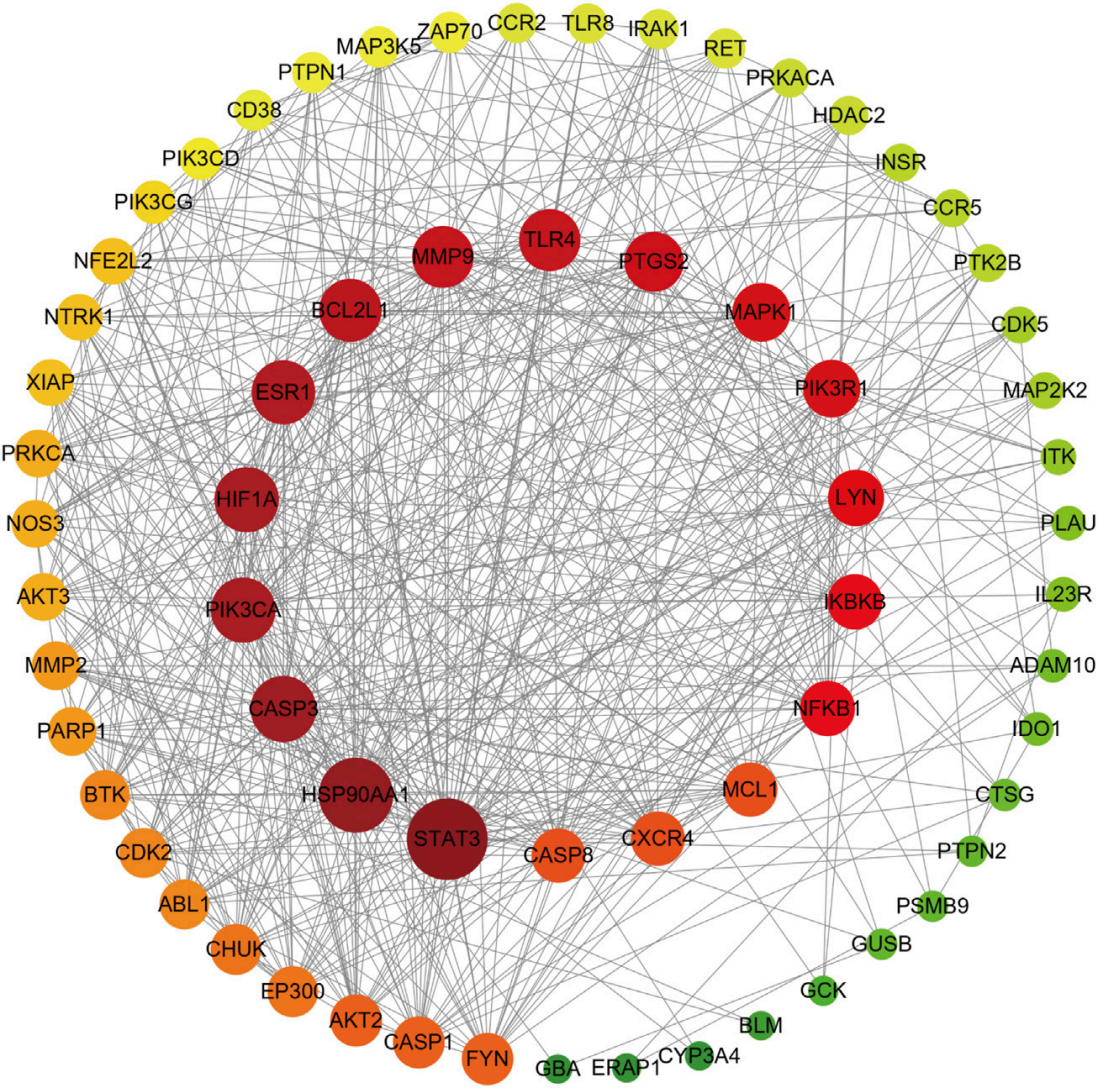
PPI network. The color and size of the nodes represent the size of the degree value, with darker red and larger nodes representing larger degree values; the inner circle represents degree values above the median.

The 66 gene targets obtained for the bioactive peptide intersecting with the disease were entered into the String database and mapped using Cytoscape 3.9.0 (Figure 5). Topological properties were analyzed by the Network Analyzer tool and those at the median degree and above were selected as key targets, as well as a total of 18 key targets were obtained and further identified by the UniProt (https://www.uniprot.org/) database (Table 2).

**TABLE 2:** 18 key target information.

Num	Target name	Gene name	UniProt ID	Degree
1	Signal transducer and activator of transcription 3	STAT3	P40763	50
2	Heat shock protein HSP 90-alpha	HSP90AA1	P07900	44
3	Caspase-3	CASP3	P42574	36
4	PI3-kinase subunit alpha	PIK3CA	P42336	35
5	Hypoxia-inducible factor 1-alpha	HIF1A	Q16665	35
6	Estrogen receptor alpha	ESR1	P03372	34
7	Bcl-2-like protein 1	BCL2L1	Q07817	33
8	Matrix metalloproteinase-9	MMP9	P14780	32
9	Toll-like receptor 4	TLR4	O00206	32
10	Prostaglandin G/H synthase 2	PTGS2	P35354	30
11	Mitogen-activated protein kinase 1	MAPK1	P28482	29
12	PI3-kinase regulatory subunit alpha	PIK3R1	P27986	28
13	Tyrosine-protein kinase Lyn	LYN	P07948	27
14	Inhibitor of nuclear factor kappa-B kinase subunit beta	IKBKB	O14920	26
15	Nuclear factor NF-kappa-B p105 subunit	NFKB1	P19838	26
16	Induced myeloid leukemia cell differentiation protein Mcl-1	MCL1	Q07820	25
17	C-X-C chemokine receptor type 4	CXCR4	P61073	25
18	Caspase-8	CASP8	Q14790	25

### 3.5 GO and KEGG enrichment analysis

**FIGURE 6:**
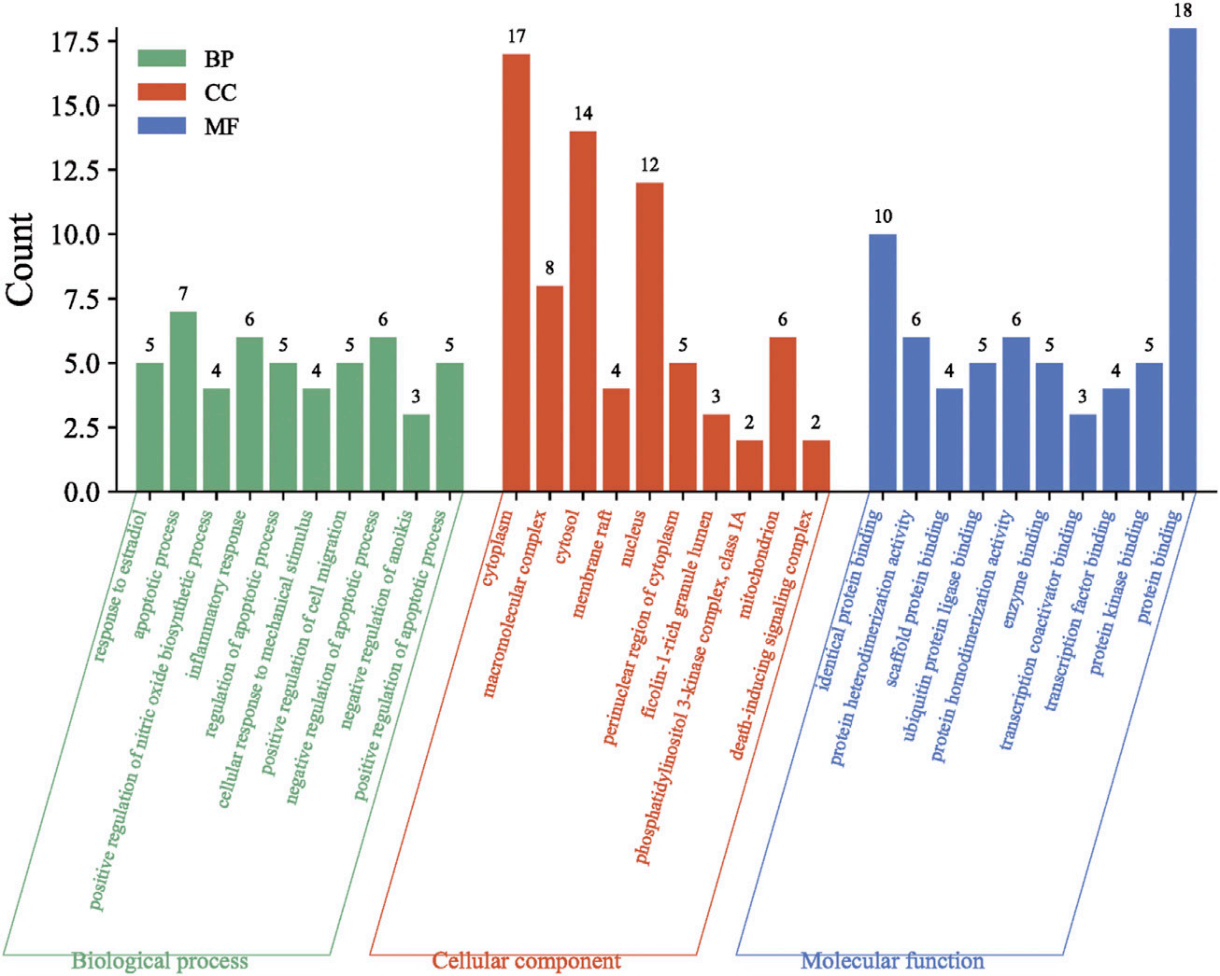
GO enrichment analysis.

The GO analysis included three components: biological process (BP), cellular component (CC) and molecular function (MF). A total of 144 biological processes or pathways were obtained by GO enrichment analysis, set *at p* < .05, of which 99 were associated with BP, 18 with CC and 27 with MF. BP mainly involves biological processes such as response to estradiol, apoptotic process, and positive regulation of nitric oxide biosynthetic process. CC mainly involves cytoplasm, macromolecular complex, cytosol and other cellular components. MF is mainly involved in identical protein binding, protein heterodimerization activity, scaffold protein binding and other functions. The top 10 GO entries were selected according to the order of *p*-value from smallest to largest and plotted in a bar chart (Figure 6).

**FIGURE 7:**
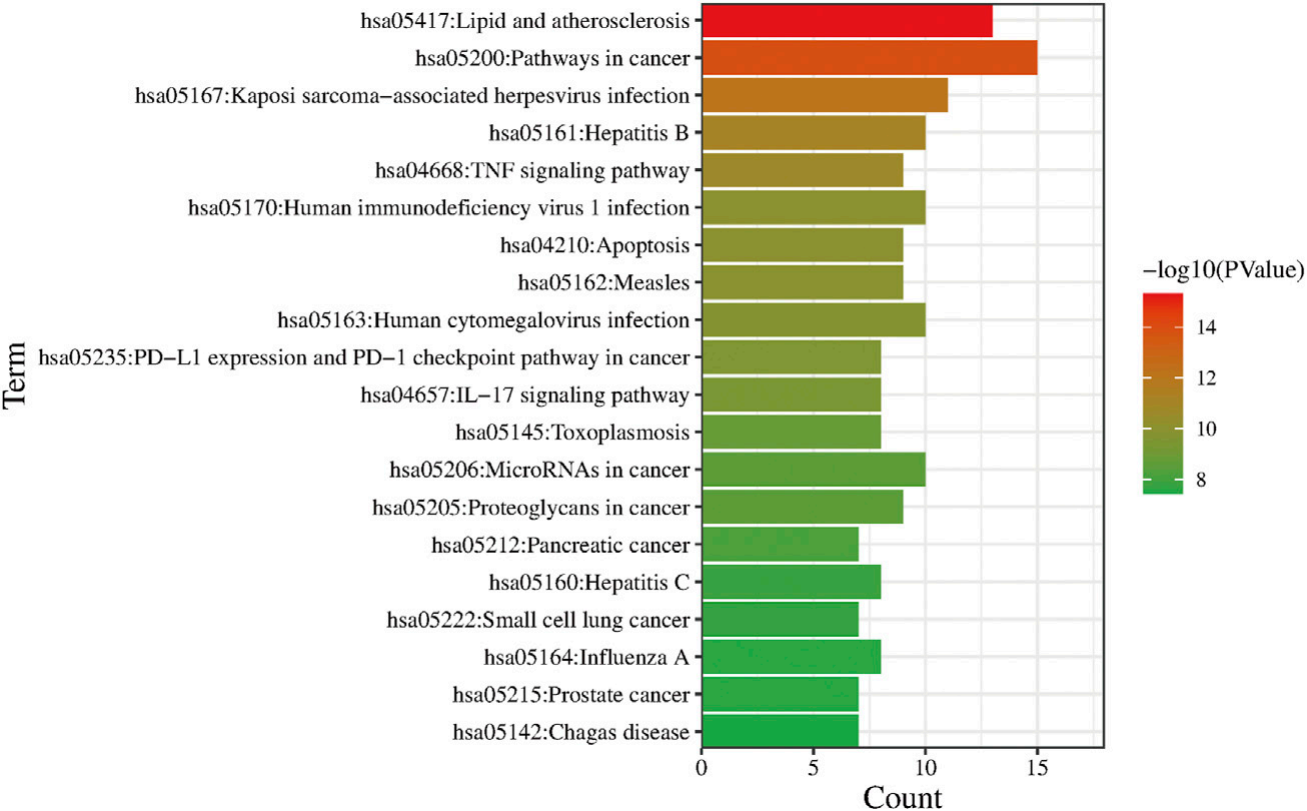
KEGG pathway enrichment analysis.

A total of 117 pathways were obtained by KEGG analysis (*p* < .05) and the main pathways are Lipid and atherosclerosis, Pathways in cancer, Kaposi sarcoma-associated herpesvirus infection, *etc.* The top 20 pathways were filtered from smallest to largest *p*-value and plotted in a bar chart (Figure 7).

### 3.6 Network construction

**FIGURE 8:**
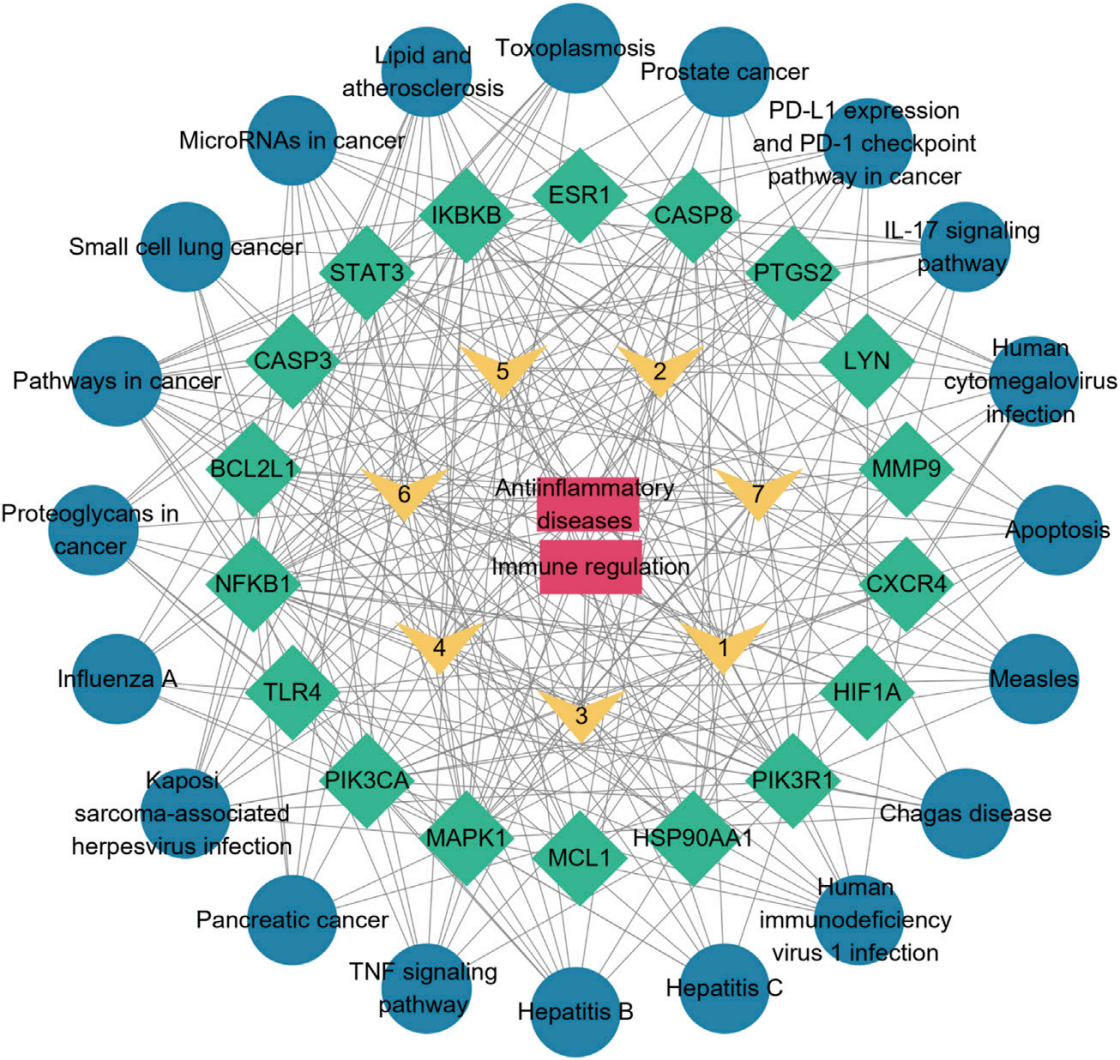
Network of “drug(bioactive peptide)-disease-target-pathway”. Red nodes represent diseases; yellow nodes represent bioactive peptides; green nodes represent key targets; blue nodes represent pathways and the line segments represent the relationship between the two.

The results of the key targets and KEGG pathway enrichment analysis of the intersection of bioactive peptides and diseases (top 20) were imported into Cytoscape 3.9.0 and constructed the “drug (bioactive peptide)-disease-target-pathway” network diagram (Figure 8).

### 3.7 Molecular docking results

**FIGURE 9:**
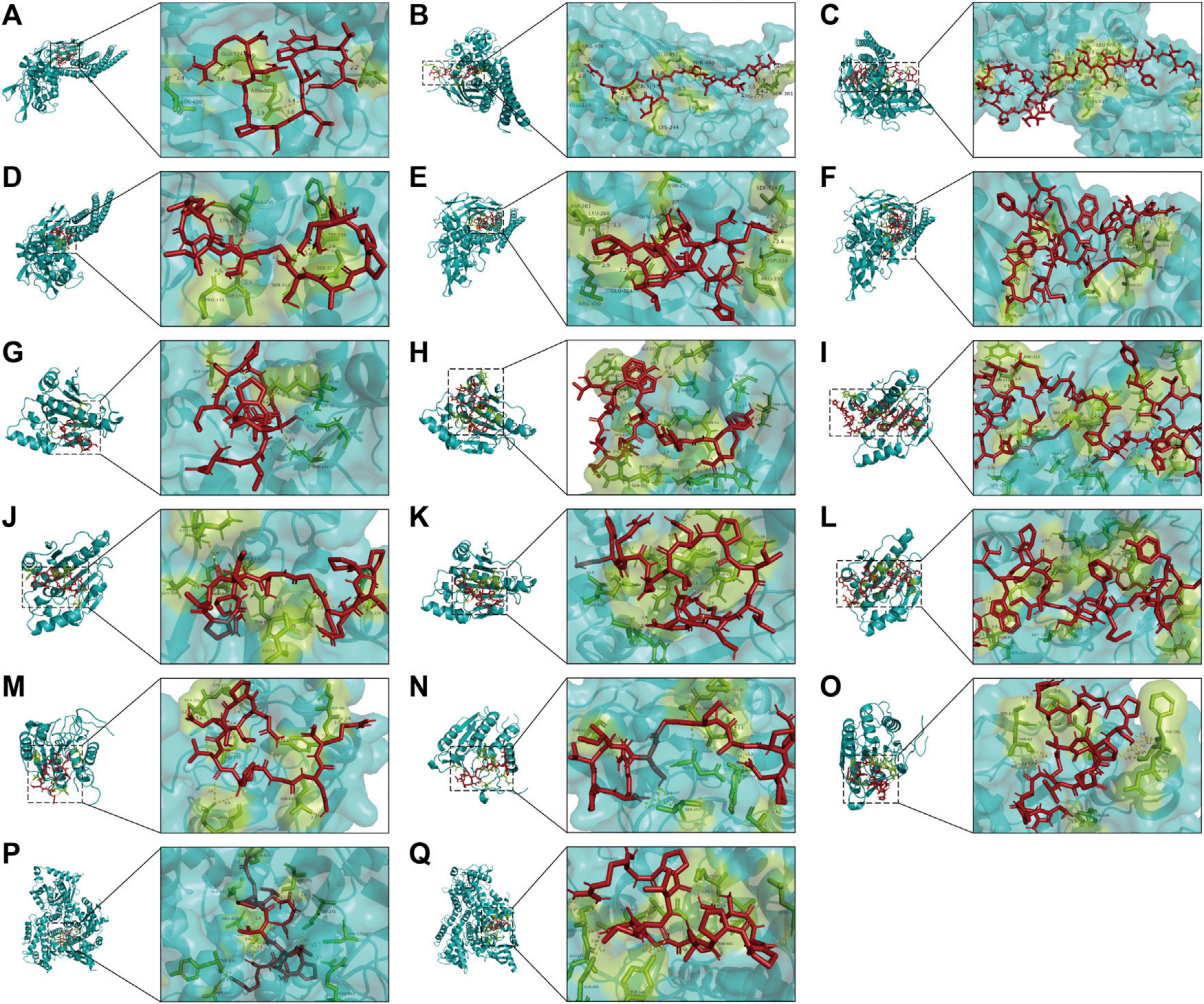
Diagram of the molecular docking pattern. **(A)** 1-STAT3; **(B)** 2-STAT3; **(C)** 4-STAT3; **(D)** 5-STAT3; **(E)** 6-STAT3; **(F)** 7-STAT3; **(G)** 1-HSP90AA1; **(H)** 3-HSP90AA1; **(I)** 4-HSP90AA1; **(J)** 5-HSP90AA1; **(K)** 6- HSP90AA1; **(L)** 7-HSP90AA1; **(M)** 1-CASP3; **(N)** 5-CASP3; **(O)** 6-CASP3; **(P)** 1-PIK3CA; **(Q)** 5-PIK3CA. Blue represents the protein; green represents the bioactive peptide binding site to the protein; red represents the active peptide.

The top four proteins STAT3, HSP90AA1, CASP3, and PIK3CA were selected as the receptors and bioactive peptides for molecular docking using Autodock vina 1.1.2 and visualized using Pymol 2.4.0 (Table3; Figure 9). Generally, affinity < −5.5 kcal/mol is considered to have better binding activity. The results showed that all molecules had affinity < −5.5 kcal/mol to the target, indicating that the bioactive peptide had better binding activity to the key target.

**TABLE 3:** Docking affinity of bioactive peptide to protein molecules.

Bioactive peptides	Gene name	PDB ID	Affinity (kcal/mol)
1	STAT3	6njs	−6.1
	HSP90AA1	5j80	−7.1
	CASP3	2dko	−.2
	PIK3CA	7jiu	−8.8
2	STAT3	6njs	−7.2
3	HSP90AA1	5j80	−7.0
4	STAT3	6njs	−6.3
	HSP90AA1	5j80	−6.2
5	STAT3	6njs	−6.2
	HSP90AA1	5j80	−7.1
	CASP3	2dko	−6.9
	PIK3CA	7jiu	−6.7
6	STAT3	6njs	−5.7
	HSP90AA1	5j80	−7.5
	CASP3	2dko	−5.9
7	STAT3	6njs	−5.8
	HSP90AA1	5j80	−7.3

### 3.8 Molecule dynamics results

The molecular docking results show that almost all of the seven bioactive peptides interact with STAT3, partly with CASP3, and the bioactive peptide 2/STAT3 and bioactive peptide 5/CASP3 have the best binding energy. Thus bioactive peptide 2/STAT3 and bioactive peptide 5/CASP3 are selected to perform molecular dynamics simulation.

**FIGURE 10:**
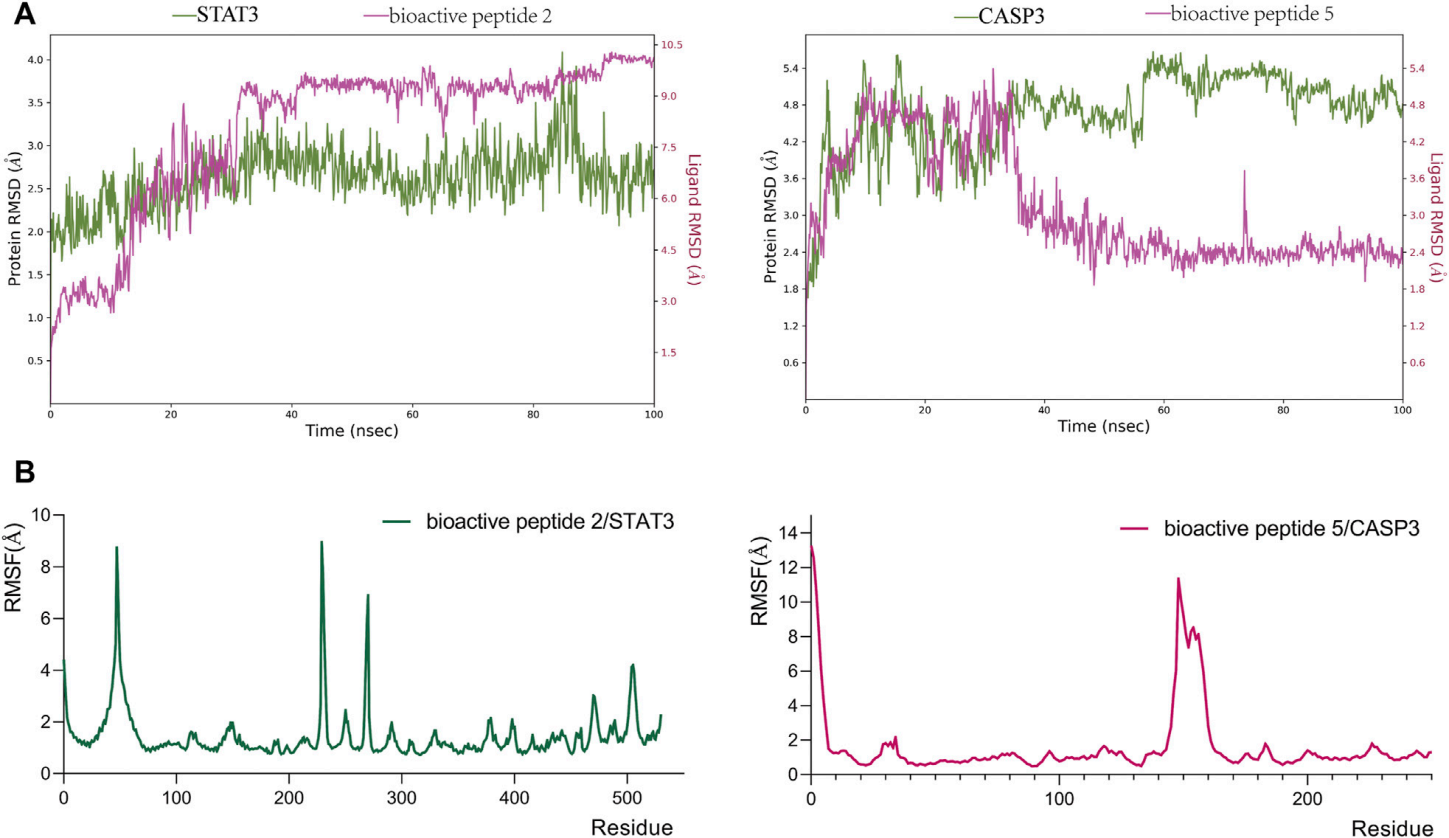
Root mean square deviation (RMSD) **(A)** and root Mean Square Fluctuation (RMSF) **(B)** during molecular dynamics simulations.

**FIGURE 11:**
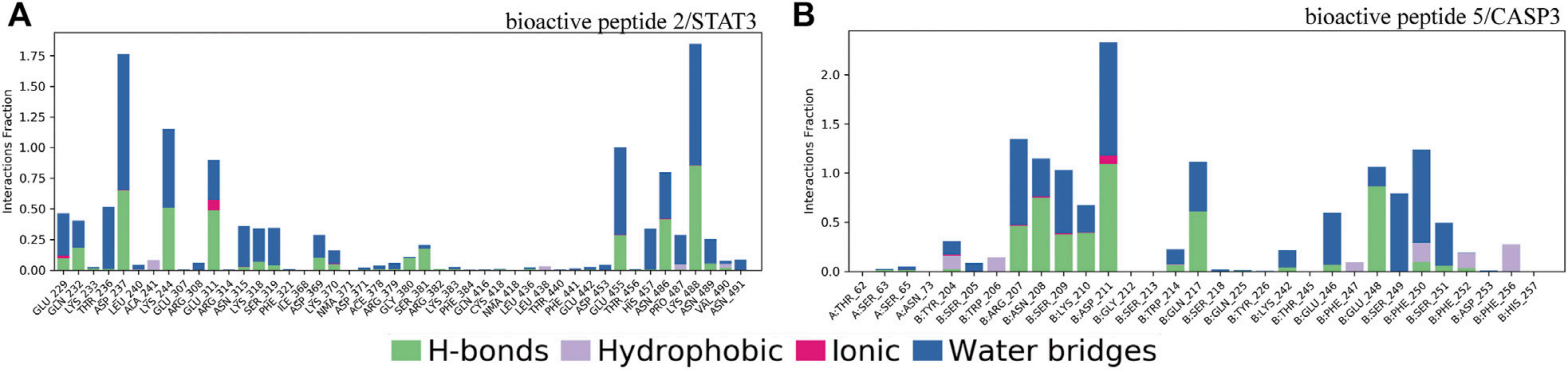
Interaction of proteins and ligands during molecule dynamics. **(A)** bioactive peptide 2/STAT3; **(B)** bioactive peptide 5/CASP3. The stacked bar charts are normalized over the course of the trajectory: for example, a value of .7 suggests that 70% of the simulation time the specific interaction is maintained.

The Root Mean Square Deviation (RMSD) can reflect the composite motion process, with large or small fluctuations representing strong or stable motion (Cao et al., 2022). The results of molecular dynamics simulation (Figure 10A) show that bioactive peptides and proteins fluctuate greatly in the early stage, move vigorously, and small fluctuations tend to stabilize in the later stage. Bioactive peptides fluctuate more than proteins and may be caused by the excessive flexibility of bioactive peptides. The root mean square fluctuation (RMSF) values can reflect the flexibility size of the protein (Chang et al., 2022). As shown in Figure 10B, the RMSFs of the two complexes are relatively low, and the RMSFs of most of the protein sequences are below 2 Å, indicating that the protein system is still relatively stable. Hydrogen Bonds (H-bonds) play a significant role in ligand binding (Cao et al., 2022). The results (Figure 11) showed that the number of hydrogen bonds in the bioactive peptide 2/STAT3 and bioactive peptide 5/CASP3 systems was more in the molecule dynamics stage.

## 4 Discussion

Bioactive peptides are considered to be a preferred alternative to drugs, being present mainly in natural foods and with low toxic effects (Shivanna and Nataraj, 2020). Moreover, dairy-derived bioactive peptides can be released during gastrointestinal digestion, food processing, or through enzymatic and bacterial fermentation and are thought to have various beneficial effects such as hypolipidemic, hypotensive, immunomodulatory, anti-inflammatory and antithrombotic effects (Marcone et al., 2017). Waqas N. Baba et al. (2021) showed that bioactive peptides from camel whey hydrolysate can assist in the treatment of hypercholesterolemia by inhibiting pancreatic lipase and cholesterol esterase. One of them, fermented camel milk from Xinjiang, is commonly consumed by Kazakhs, Mongolians, and other ethnic groups and contains unique microflora (Ghosh et al., 2019). Fermented camel milk is rich in probiotics (Rabab M.H.A. Madany, 2017), and many of probiotics’ health benefits and disease alleviation abilities are achieved through immune induction (Varsha et al., 2021). Research has shown that probiotics can enhance the innate immunity of the organism by upregulating the number of immunoglobulins and antimicrobial proteins in the body and increasing the activity of phagocytes and natural killer cells after normal host cell colonization; they can enhance the acquired immunity of the organism by increasing the function of antigen-presenting B cells and T lymphocytes (Di Pierro et al., 2014). In this work, we used network pharmacology and molecular docking approach to elucidate the molecular biological mechanisms of immune modulation by bioactive peptides.

In this study, the KEGG study shows lipid and atherosclerosis, as well as pathways in cancer, are the two most enriched pathways. Atherosclerosis is a chronic inflammatory disease that is prevented and treated through the interplay of lipid metabolism and inflammatory responses (Liu et al., 2014). The development of cancer is usually accompanied by inflammation and cancer development and metastasis can be inhibited by treating or controlling inflammation and blocking the production of related factors (e.g., TNF-α, IL-6, IL-17, etc.) (Landskron et al., 2014). The STAT3 signaling pathway is a major intrinsic pathway in cancer inflammation as it is frequently activated in malignant cells and can induce a large number of inflammations (Johnson et al., 2018). STAT3 plays a dual role in tumor inflammation and immunity by promoting the pro-tumor inflammatory pathway of nuclear factor-κb (NF-κb) and interleukin-6 (IL-6)-GP130-Janus kinase (JAK) and by inhibiting STAT1 and NF-κb-mediated Th1 antitumor immune responses (Yu et al., 2009; Gharibi et al., 2020). It has been shown that in the immune system, receptors for most cytokines expressed by lymphocytes such as IL-6, IL-10 and interferons (IFNs) can also activate STAT3 (Deenick et al., 2018). Heat shock proteins (HSP) have different effects on the immune system and they can stimulate or improve the immune response (Androvitsanea et al., 2021). Newly formed HSP90AA1 can be secreted into the extracellular environment and the nucleus, stimulating the formation of immune memory and participating in tumor formation (Guo et al., 2022). The apoptosis-associated gene CASP3 causes nuclear DNA breaks during apoptosis and CASP3 is also involved in MAPK signaling pathways and inflammatory responses (Ma et al., 2021). PIK3CA regulates cell growth and apoptosis by participating in the PI3K/AKT signaling pathway (Shi et al., 2021). GO and KEGG analyses have shown that the apoptotic process plays an important role. Aberrant apoptosis leads to excessive cell clearance or prolonged survival and is therefore involved in the pathogenesis of many immune diseases through the regulation of aberrant apoptosis (Sankari et al., 2015). According to the aforementioned findings, the bioactive peptides’ immunomodulatory and anti-inflammatory effects can be attributed to a variety of biological mechanisms and signaling pathways. The molecular docking results show that the seven bioactive peptides bind well to the first four key targets, demonstrating the reliability of the network pharmacology predictions. And the binding of bioactive peptides to proteins was also found to be stable after molecular dynamics simulations.

This study explored the immunomodulatory and anti-inflammatory mechanisms of bioactive peptides extracted from fermented camel milk in Xinjiang based on network pharmacology and molecular docking. By speculating on the targets and pathways of action of bioactive peptides extracted from fermented camel milk in Xinjiang, this paper provides theoretical support for future experiments to be conducted. It also provides some references for the future development of fermented camel milk in Xinjiang.

## Data Availability

The datasets presented in this study can be found in online repositories. The names of the repository/repositories and accession number(s) can be found in the article/Supplementary Material.
